# Radiopaque Strontium Fluoroapatite Glass-Ceramics

**DOI:** 10.3389/fbioe.2015.00149

**Published:** 2015-10-13

**Authors:** Wolfram Höland, Marcel Schweiger, Marc Dittmer, Christian Ritzberger

**Affiliations:** ^1^Research and Development, Inorganic Chemistry, Technical Fundamentals, Ivoclar Vivadent AG, Schaan, Liechtenstein

**Keywords:** glass-ceramics, phosphate crystals, nucleation and crystallization, crystal growth, Ostwald ripening, radiopacity, dental restoration, biomaterial

## Abstract

The controlled precipitation of strontium fluoroapatite crystals was studied in four base glass compositions derived from the SiO_2_–Al_2_O_3_–Y_2_O_3_–SrO–Na_2_O–K_2_O/Rb_2_O/Cs_2_O–P_2_O_5_–F system. The crystal phase formation of these glasses and the main properties of the glass-ceramics, such as thermal and optical properties and radiopacity were compared with a fifth, a reference glass-ceramic. The reference glass-ceramic was characterized as Ca-fluoroapatite glass-ceramic. The four strontium fluoroapatite glass-ceramics showed the following crystal phases: (a) Sr_5_(PO_4_)_3_F – leucite, KAlSi_2_O_6_, (b) Sr_5_(PO_4_)_3_F – leucite, KAlSi_2_O_6_, and nano-sized NaSrPO_4_, (c) Sr_5_(PO_4_)_3_F – pollucite, CsAlSi_2_O_6_, and nano-sized NaSrPO_4_, and (d) Sr_5_(PO_4_)_3_F – Rb-leucite, RbAlSi_2_O_6_, and nano-sized NaSrPO_4_. The proof of crystal phase formation was possible by X-ray diffraction. The microstructures, which were studied using scanning electron microscopy, demonstrated a uniform distribution of the crystals in the glass matrix. The Sr-fluoroapatites were precipitated based on an internal crystallization process, and the crystals demonstrated a needle-like morphology. The study of the crystal growth of needle-like Sr-fluoroapatites gave a clear evidence of an Ostwald ripening mechanism. The formation of leucite, pollucite, and Rb-leucite was based on a surface crystallization mechanism. Therefore, a twofold crystallization mechanism was successfully applied to develop these types of glass-ceramics. The main focus of this study was the controlled development of glass-ceramics exhibiting high radiopacity in comparison to the reference glass-ceramic. This goal could be achieved with all four glass-ceramics with the preferred development of the Sr-fluoroapatite – pollucite-type glass-ceramic. In addition to this main development, it was possible to control the thermal properties. Especially the Rb-leucite containing glass-ceramic showed the highest coefficient of thermal expansion (CTE). These glass-ceramics allow optical properties, especially the translucency and color, to be tailored to the needs of biomaterials for dental applications. The authors conclude that it is possible to use twofold crystallization processes to develop glass-ceramic biomaterials featuring different properties, such as specific radiopacity values, CTEs, and optical characteristics.

## Introduction

The precipitation of fluoroapatites, in glass-ceramics using the methods of controlled nucleation and crystallization of glasses, is well known. Fluoroapatite glass-ceramics were reported by Clifford and Hill (1996) and Moisescu et al. (1999). Furthermore, bioactive glass-ceramics, which bond to living bone, contain fluoroapatite crystals. Such a product was developed by Kokubo (1991) who applied the method of twofold nucleation to create a fluoroapatite–wollastonite glass-ceramic. Dejneka and Pinckney (1998) developed fluoroapatite glass-ceramics with special optical properties. All these different types of apatite glass-ceramics were discussed and described on the basis of their chemical nature and microstructure formation by Höland and Beall (2012). Höland et al. (2004) reported on the formation of Sr-fluoroapatite glass-ceramics with the possibility of forming a primary crystal phase of NaSrPO_4_. Moreover, van‘t Hoen et al. (2007) also succeeded in growing different types of siliceous oxyapatites in glass-ceramics. The substitution of different ions in fluoroapatite-type glass-ceramics was studied by Chen et al. (2014) and Hill et al. (2004, 2010) with the aim of developing strontium fluoroapatite and chlorapatite-type glass-ceramics. The development of an alumosilicate glass-ceramic of the pollucite-type (CsAlSi_2_O_6_) was achieved by Beall and Rittler (1982). Twofold nucleation and crystallization of both leucite (KAlSi_2_O_6_) and Ca-fluoroapatite (Ca_5_(PO_4_)_3_F) was reported by Höland et al. (1994, 2000, 2004, 2008). The growth of needle-like Ca_5_(PO_4_)_3_F crystals was discovered as an Ostwald ripening process by Müller et al. (1999) and Höland et al. (2000) and in a different chemical system by Höche et al. (2001).

Radiopaque glasses are known as glass ionomer cements in dentistry (Tsuge, 2009). Also, glass-ceramics were developed as radiopaque materials in inorganic–organic composites as dental fillers. Müller (1973) succeeded in developing such a glass-ceramic with high contents of La_2_O_3_. The purpose of the present study was to develop a glass-ceramic with high radiopacity and additional properties of controlled translucency and special coefficients of thermal expansion.

Fundamental research on the nucleation process of phosphosilicate glass-ceramics showed the special function of glass-in-glass phase separation. The basis for these processes of controlled immiscibility in glasses on the way to forming glass-ceramics via controlled nucleation and crystallization was established by Vogel (1963, 1985) and discussed in detail by Kreidl (1983). This phenomenon of microstructure formation provided a basis for the development of many different types of glass-ceramics. Building on these fundamentals, Vogel and Höland (1987) developed a phosphosilicate base glass consisting of three glassy phases: two droplet phases and a glass matrix. By annealing this glass in a controlled way, the precipitation of two crystal phases was possible: the mica-type and the calcium fluoroapatite crystals. This established the basis for the development of a bioactive biomaterial for bone substitution.

Nevertheless, the authors of this manuscript will neither focus on bioactive glass-ceramics nor on glass-ceramics for technical application. Their main aim is to develop glass-ceramics as biomaterials for applications in restorative dentistry. In the development of new dental restorative biomaterials (e.g., materials for dental inlays, onlays, or crowns), new properties are explored, for example, how to make it easier for dental practitioners to detect the interface between the biomaterial and the natural tooth very precisely. In order to enable such a distinction, the radiopacity of the biomaterial could be increased, for example.

For this purpose, the authors selected four glass compositions derived from the SiO_2_–Al_2_O_3_–Y_2_O_3_–SrO–Na_2_O–K_2_O/Rb_2_O/Cs_2_O–P_2_O_5_–F system. They studied the processes of nucleation and crystallization of strontium fluoroapatite and additional crystal phases designed the microstructure of the glass-ceramics and determined the main properties of these types of glass-ceramics with the aim of controlling the radiopacity and to develop additional properties (optical, thermal).

In addition to these four selected glass-ceramics, a fifth glass-ceramic composition was prepared, which was free of Y_2_O_3_, SrO, Cs_2_O, and Rb_2_O. However, this composition contained CaO to allow the precipitation of calcium fluoroapatite, Ca_5_(PO_4_)_3_F crystals. This experimental, not commercial glass-ceramic was used as a reference material, primarily for the radiopacity study.

## Materials and Methods

### Processing and chemical compositions

Four compositions of base glasses were selected to develop the new glass-ceramics. As mentioned, the glasses were derived from the chemical SiO_2_–Al_2_O_3_–Y_2_O_3_–SrO–Na_2_O–K_2_O/Rb_2_O/Cs_2_O–P_2_O_5_–F system, but with additives of small amounts of ZrO_2_, TiO_2_, CeO_2_, B_2_O_3_, and Li_2_O. The chemical compositions are shown in Table 1.

**Table 1:** **Chemical composition in wt.-% and (mol.-%) of the base glasses used to develop glass-ceramics**.

	1	2	3	4	5 (Reference)
SiO_2_	47.0 (58.7)	48.6 (59.4)	44.6 (60.4)	49.0 (61.1)	59.3 (66.0)
Al_2_O_3_	12.2 (9.0)	12.6 (9.1)	11.3 (9.0)	12.4 (9.1)	13.0 (8.5)
Y_2_O_3_	9.2 (3.1)	6.2 (2.2)	0.4 (0.1)	0.4 (0.1)	
La_2_O_3_			1.1 (0.3)		
CaO					1.5 (1.8)
SrO	7.5 (5.5)	7.8 (5.5)	10.1 (7.9)	8.1 (5.9)	
ZnO					1.6 (1.3)
Na_2_O	8.3 (10.1)	8.6 (10.2)	6.4 (8.4)	8.3 (10.0)	8.2 (8.9)
K_2_O	9.4 (7.5)	9.7 (7.6)	3.0 (2.6)	3.4 (2.7)	9.0 (6.4)
P_2_O_5_	3.4 (1.8)	3.4 (1.8)	3.1 (1.8)	3.3 (1.7)	0.4 (0.2)
F	0.6 (2.4)	0.6 (2.3)	0.6 (2.6)	0.6 (2.4)	0.9 (3.2)
Cs_2_O			17.1 (4.9)		
Rb_2_O				12.0 (4.8)	
ZrO_2_	0.8 (0.5)	0.9 (0.5)	0.7 (0.5)	0.9 (0.6)	3.8 (2.1)
TiO_2_	0.2 (0.2)	0.2 (0.2)	0.2 (0.2)	0.2 (0.2)	1.4 (1.2)
CeO_2_	0.9 (0.4)	0.9 (0.4)	0.9 (0.4)	0.9 (0.4)	0.8 (0.3)
B_2_O_3_	0.3 (0.3)	0.3 (0.3)	0.3 (0.4)	0.2 (0.2)	0.1 (0.1)
Li_2_O	0.2 (0.5)	0.2 (0.5)	0.2 (0.5)	0.3 (0.8)	

As a reference to these four compositions, a fifth base glass was used, which was derived from the chemical SiO_2_–Al_2_O_3_–CaO–Na_2_O–K_2_O–P_2_O_5_–F system with additives of small amounts of ZnO, ZrO_2_, TiO_2_, CeO_2_, and B_2_O_3_ (Table 1). The basis for the design of the chemical compositions follows the ideas:

- To study the influence of an increase of Y_2_O_3_ in glass-ceramics 1 and 2 over the reference sample 5.
- To investigate the influence of relatively high contents of Cs_2_O (glass 3) and Rb_2_O (glass 4) over glass-ceramics 5, 1 and 2.
- The additives of many other components to the glass-ceramics (especially the alkali ions) were necessary to reach the best melting conditions for the base glasses and an optimum of sintering to produce dense powder compacts.

The glasses were molten based on the raw materials of SiO_2_, TiO_2_, ZrO_2_, CeO_2_, Y_2_O_3_, aluminum-oxyhydroxy hydrate, carbonates of sodium, potassium, rubidium, cesium, strontium, calcium, magnesium, lithium, aluminum metaphosphate, sodium fluoride, and boron oxide hydrate. The melt was carried out in a platinum crucible and the conditions used were 1600°C with a melting time of 2 h.

The main aim of this study was to produce glass-ceramics via the powder compact route, which has been discussed in detail by Höland and Beall (2012). The reason for applying this method was the ability to take advantage of a twofold nucleation and crystallization process involving both surface and internal mechanisms. This is the same methodology which is applied to produce leucite-fluoroapatite glass-ceramics (Höland et al., 2000). After melting the glasses, the process was started by pouring the hot melt into water and drying the produced glass frits. The glass frits were investigated by X-ray diffraction (XRD). Next, the frits were ground by ball milling to a grain size of <90 μm to a mean grain size of approximately 35 μm. Subsequently, the glass granules were heat treated at 900°C, and in a separate sample preparation to 1000°C for 1 h. After these heat treatments, all the samples were ground again to a grain size of approximately 35 μm. This process was followed by the final process of powder compact preparation. This was carried out in the Programat^®^ P700 furnace (Ivoclar Vivadent AG) under a vacuum of 20–25 mbar and at a heating rate of 40 K/min at different temperatures:

- Disks with a diameter of 20 mm and a thickness of 2 mm for scanning electron microscopy (SEM) studies and optical measurements, and 1 mm thickness for determining the radiopacity:
  - glass-ceramic 1: 960°C for 1 min
  - glass-ceramic 2: 940°C for 1 min
  - glass-ceramic 3: 1150°C for 1 min
  - glass-ceramic 4: 1030°C for 1 min
  - glass-ceramic 5: 960°C for 1 min
- Bars (27 mm × 5 mm × 4 mm) for measuring the coefficients of thermal expansion:
  - glass-ceramic 1: 940 and 960°C for 1 min
  - glass-ceramic 2: 920 and 940°C for 1 min
  - glass-ceramic 3: 1130 and 1150°C for 1 min
  - glass-ceramic 4: 1010 and 1030°C for 1 min
  - glass-ceramic 5: 940 and 960°C for 1 min.

These different temperatures were selected from a great variety of trails to reach the best condition of dense powder compact preparation. In order to study the microstructure of the base glasses by SEM and to investigate the internal crystal growth mechanism (without additional surface crystallization), however, monolithic samples were prepared. Therefore, in a separate processing step, portions of the glass melt were cast on a copper plate and cooled to room temperature, with a cooling range of 3–5 K/min from the *T*_g_ range to room temperature. The surface of the samples was removed by grinding and the volume was investigated by SEM and XRD.

These monolithic samples were also used to study the crystal growth of Sr-fluoroapatite (Sr_5_(PO_4_)_3_F). This procedure allowed the analysis of the crystallization mechanism of the internally formed crystals without the influence of the surface crystallization mechanism. However, in order to compare these results with the growth of needle-like Ca_5_(PO_4_)_3_F in a different chemical glass-ceramic system (Höland et al., 1994, 2000), the growth of crystals was studied at 1000°C. For this purpose, the glass-ceramic No. 2 was annealed at 1000°C for different times: 30, 60, 120, 240 and 480 min. The resulting microstructures were studied with SEM, and the crystal numbers, crystal lengths, and crystal diameters were determined and plotted as functions of time.

### Methods

The chemical compositions of all the base glasses were analyzed by X-ray fluorescence (XRF) using Tiger S8, Bruker AXS, Karlsruhe, Germany with regard to the main constituents. However, Li_2_O and B_2_O_3_ were determined according to atomic absorption spectrometry (AAS) using HT-200, Varian, Darmstadt, Germany. Fluorine was analyzed using the fluoride selective electrode method. All these compositions are shown in Table 1.

The determination of the *T*_g_ values of the glasses was studied by means of differential scanning calorimetry (DSC). These DSC investigations of glass grains were carried out with the apparatus STA 449 F3 (Netzsch, Germany) using a heating rate of 10 K/min and N_2_ atmosphere.

The microstructures of the monolithic base glasses and the powder compact glass-ceramic samples were studied by means of scanning electron microscopic, SEM, investigations of etched samples. Mainly HF etching (aqueous 3% HF solution, for 10 s) was used in addition to etching with H_3_PO_4_. The SEM investigations were carried out with the apparatus Supra 40VP (Carl Zeiss, Oberkochen, Germany).

The crystal phase was successfully analyzed by XRD, with the D8 Advance device using a LYNXEYE detector and Cu–K_α_ radiation (Bruker, Karlsruhe, Germany).

Thermal properties as *T*_g_ values of the glasses and the coefficients of thermal expansion (CTEs) of the powder compact glass-ceramics were determined. The *T*_g_ values were established as important characteristics (fixed point) of glasses using DSC STA 449 Netzsch (Selb, Germany). The *T*_g_ points were determined as onset points of the DSC peaks. The CTE parameters were analyzed by using the apparatus TA Instruments DIL Type 803 (Hüllhorst, Germany) (formerly Bähr).

The optical properties in terms of L*, a*, b* values according to DIN5033 and DIN6174 were established with a CM-3700d Spectrometer (Konica Minolta). The optical values of CR were determined according the British Standard 5612. A value of 100 would represent a 100% opaque product and the value 0 a material that is transparent in visible light.

The radiopacity was characterized on sintered samples with a diameter of 20 mm and a thickness of 1 mm. The specific radiopacity was analyzed according to EN ISO 4049. The radiopacity values were calculated on the basis of the gray shade of the radiograph in comparison to an aluminum standard. The radiographs were taken with a Heliodent^Plus^ X-ray system (Sirona, Bensheim, Germany) on a Carestream CS7600 No. 2 imaging plate (Carestream Dental, Atlanta, GA, USA). The evaluation of the specific gray shade was done with the Adobe^®^ Photoshop software (Adobe^®^ Systems, San José, CA, USA).

## Results

### Base glasses

**Figure 1:**
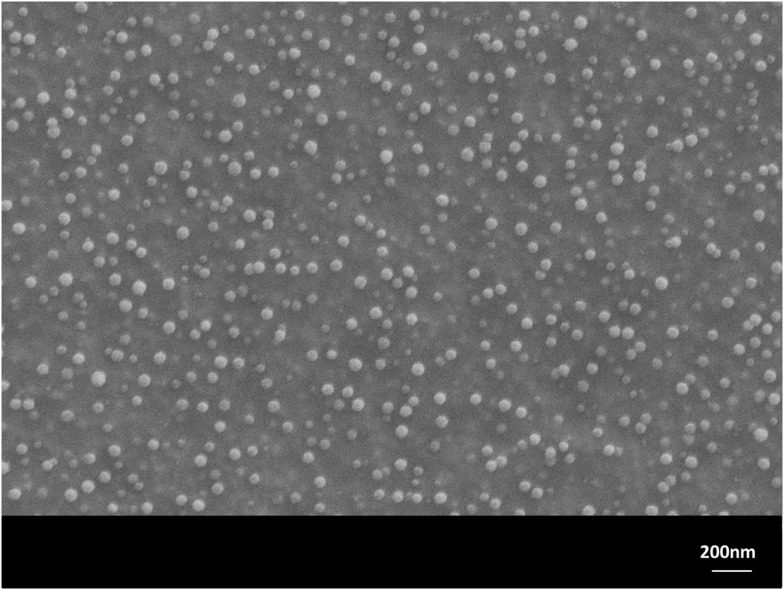
**Microstructure of glass No. 2 after 1 h heat treatment at 600°C**. Sr_5_(PO_4_)_3_F (with minor NaSrPO_4_) spherical crystals of nanometer size are visible. Monolithic sample, polished surface, HF etched. SEM.

The monolithic base glasses No. 1 and 5 were optically transparent in visible light, while the other glasses No. 2–4 showed an opalescent effect. But XRD studies of all four monolithic glass samples, showed the formation of primary crystal phases, precipitated during cooling of the glass melts. The microstructures of these glasses No. 1–4 were characterized by nano-sized spherical crystals of different types: nano-sized Sr_5_(PO_4_)_3_F crystals in glasses No. 1 and 2 (with a very minor content of NaSrPO_4_ in glass No. 2) and nano-sized NaSrPO_4_ crystals in glasses No. 3 and 4. The crystals were analyzed by XRD. The general formation of NaSrPO_4_ was described by Bredig (1942). Figure 1 shows the approximately 50 nm crystals [mainly Sr_5_(PO_4_)_3_F with minor NaSrPO_4_], which have precipitated in glass No. 2 in isolated form in the glass matrix. Heat treating these samples at 600 or 700 or 800°C for 1 h did not change the microstructure.

Using a H_3_PO_4_ etching procedure, the crystals were etched away and holes of approximately 50 nm were visible in the SEM micrograph. The glass frits No. 1–4, which were produced by pouring the hot melt into water (representing a fast cooling rate), were also investigated by XRD. All these glasses contained nano-sized crystals. Glasses No. 1 and 2 showed Sr_5_(PO_4_)_3_F crystals (glass two with a minor content of NaSrPO_4_) and glasses No. 3 and 4 showed NaSrPO_4_ crystals. But the base glass No. 5, the reference sample, was a crystal-free material, and was produced either as a monolithic sample or a frit.

**Figure 2:**
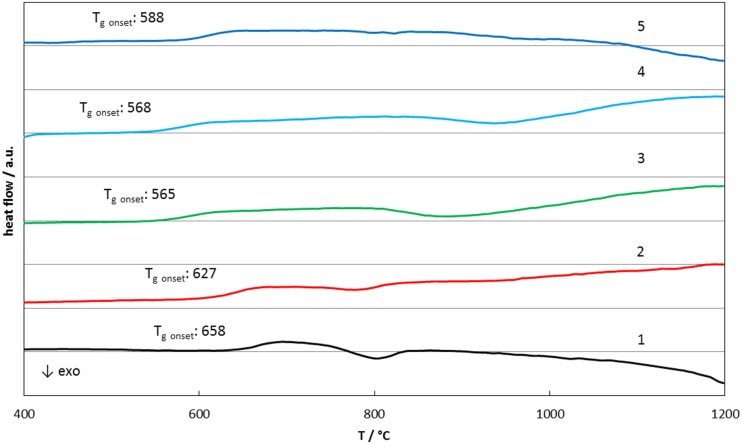
**Differential scanning calorimetry results of glasses 1–5**.

The glass frits were also used to determine the *T*_g_ values of the glasses, that is the glass matrices with the nano-sized crystals. These characteristic *T*_g_ values were detected between 570 and 670°C (glass No. 1: 658°C, glass No. 2: 627°C, glass No. 3: 565 No. 4: 568°C, glass No. 5: 588°C, all with an error margin of ±3 K) according to Figure 2. These values gave hints for further heat treatment cycles to control the crystallization processes in a range from 800 to 1000°C.

### Microstructure formation of glass-ceramics

**Figure 3:**
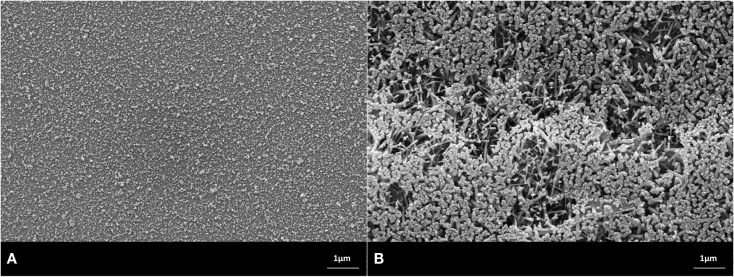
**Microstructures of heat-treated glass frits of composition No. 1, processed at (A) 900°C/h, (B) 1000°C/h. Fractured surface, HF etched sample (10 s, 3% HF)**. SEM. **(A)** Glass-ceramic with the crystal phase of Sr_5_(PO_4_)_3_F. **(B)** Glass-ceramic containing crystals of Sr_5_(PO_4_)_3_F and KAlSi_2_O_6_.

**Figure 4:**
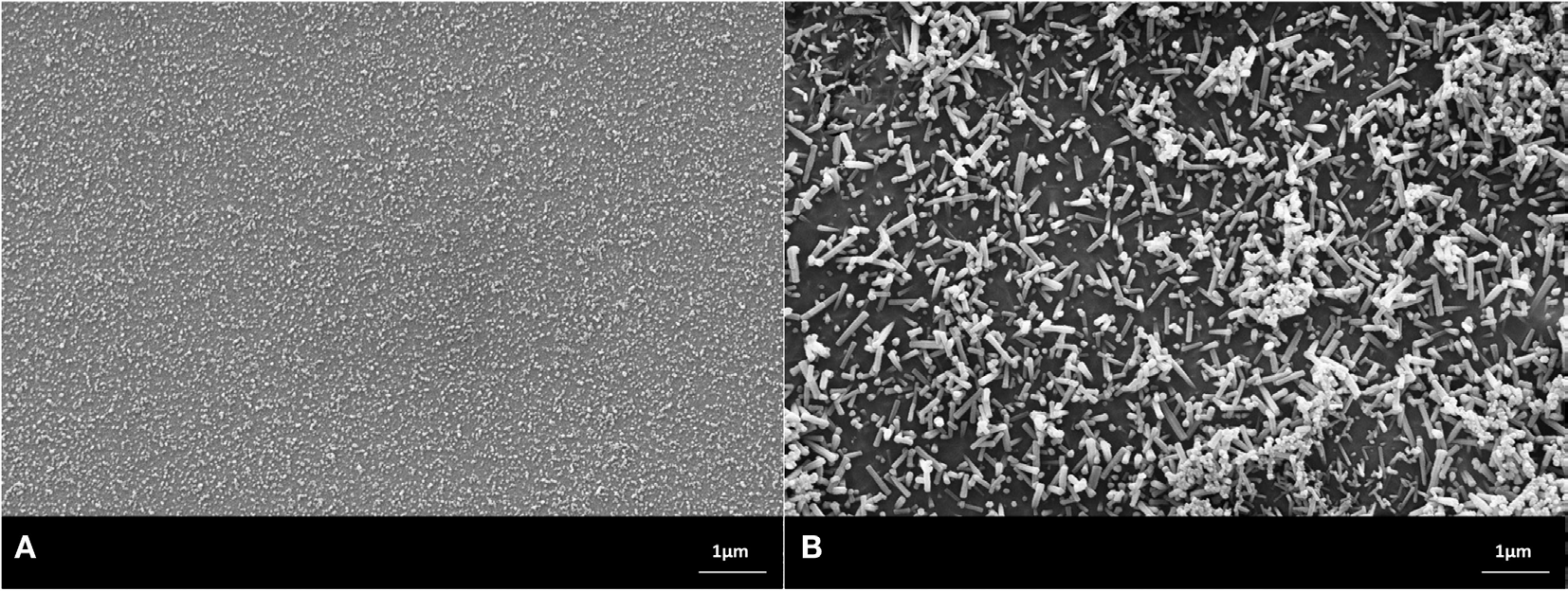
**Microstructures of heat-treated glass frits of composition No. 2, processed at (A) 900°C/h, (B) 1000°C/h**. Fractured surface, HF etched sample (10 s, 3% HF). SEM. **(A)** Glass-ceramic with the crystal phase of Sr_5_(PO_4_)_3_F and a minor content of NaSrPO_4_. **(B)** Glass-ceramic containing crystals of Sr_5_(PO_4_)_3_F, KAlSi_2_O_6_, and a minor content of NaSrPO_4_.

**Figure 5:**
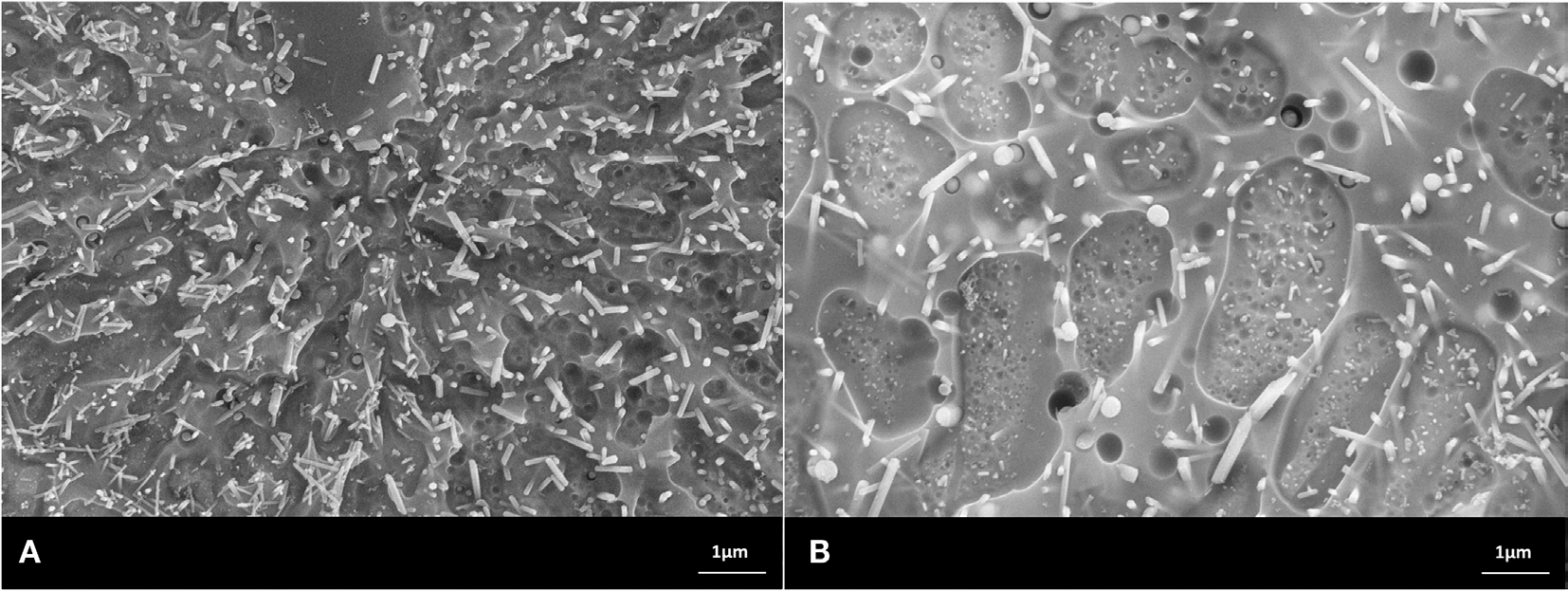
**Microstructures of heat-treated glass frits of composition No. 3, processed at (A) 900°C/h, (B) 1000°C/h**. Fractured surface, HF etched sample (10 s, 3% HF). SEM. **(A,B)** Glass-ceramics with crystal phases of Sr_5_(PO_4_)_3_F, CsAlSi_2_O_6_, and NaSrPO_4_.

**Figure 6:**
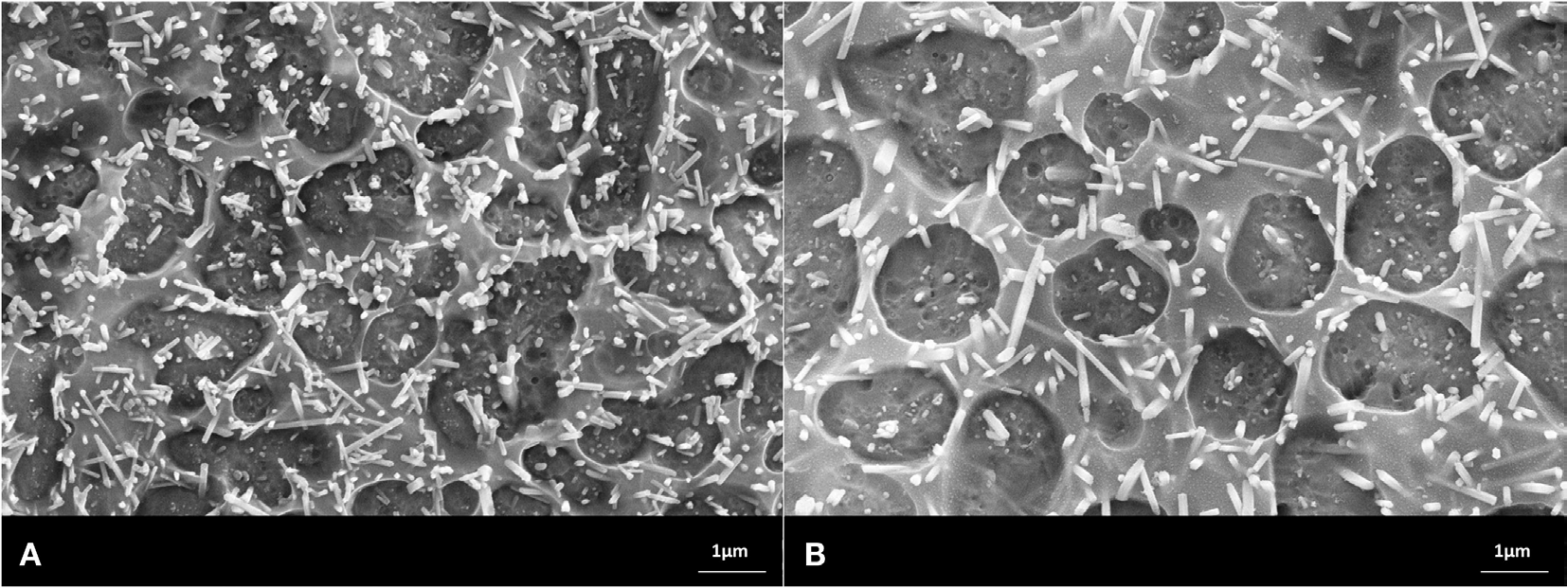
**Microstructures of heat-treated glass frits of composition No. 4, processed at (A) 900°C/h, (B) 1000°C/h**. Fractured surface, HF etched sample (10 s, 3% HF). SEM. **(A,B)** Glass-ceramics with crystal phases of Sr_5_(PO_4_)_3_F, RbAlSi_2_O_6_, and NaSrPO_4_.

**Figure 7:**
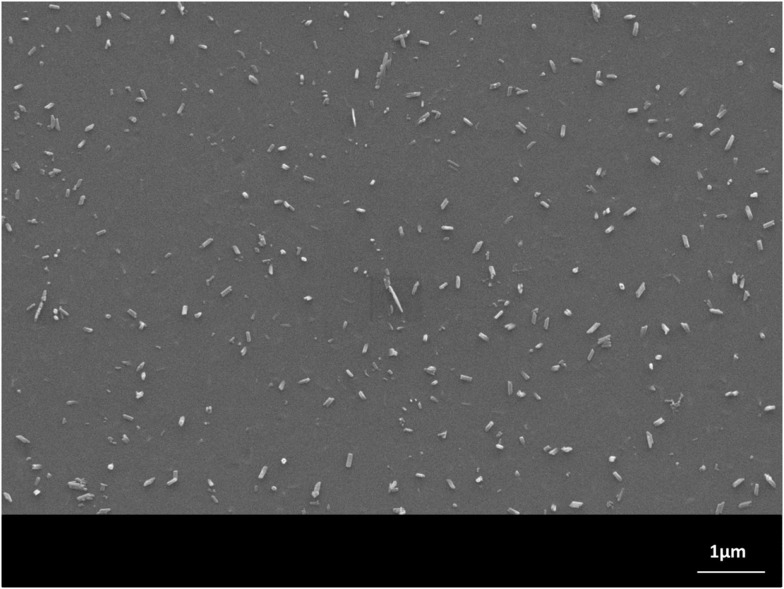
**Microstructures of heat-treated glass frits of composition No. 5, processed at 900°C/h**. Fractured surface, HF etched sample (10 s, 3% HF). SEM. The glass-ceramic contains needle-like Ca_5_(PO_4_)_3_F crystals.

Scanning electron microscopy studies allowed the characterization of the design of the powder compact glass-ceramics and XRD enabled the determination of the type of crystal phases. The main crystal phase formation and the design of the microstructure of the glass-ceramics are shown in Figures 3–7.

The microstructures shown in the SEMs in Figures 3–7 have to be interpreted on the basis of the preparation method of HF etching and in correlation with the XRD studies. HF treatment of these types of glass-ceramics results in the etching of the SiO_2_-rich glass matrix and especially the interface of the glass surrounding the alumosilicate or phosphate crystals. With regard to the alumosilicates, therefore, the interface between the crystals and the glass matrix was etched away and as a consequence the crystals fell out of the surface of the glass-ceramic. As a result, holes were created in the surface of the sample, and these holes represent the size of the alumosilicate crystals.

**Figure 8:**
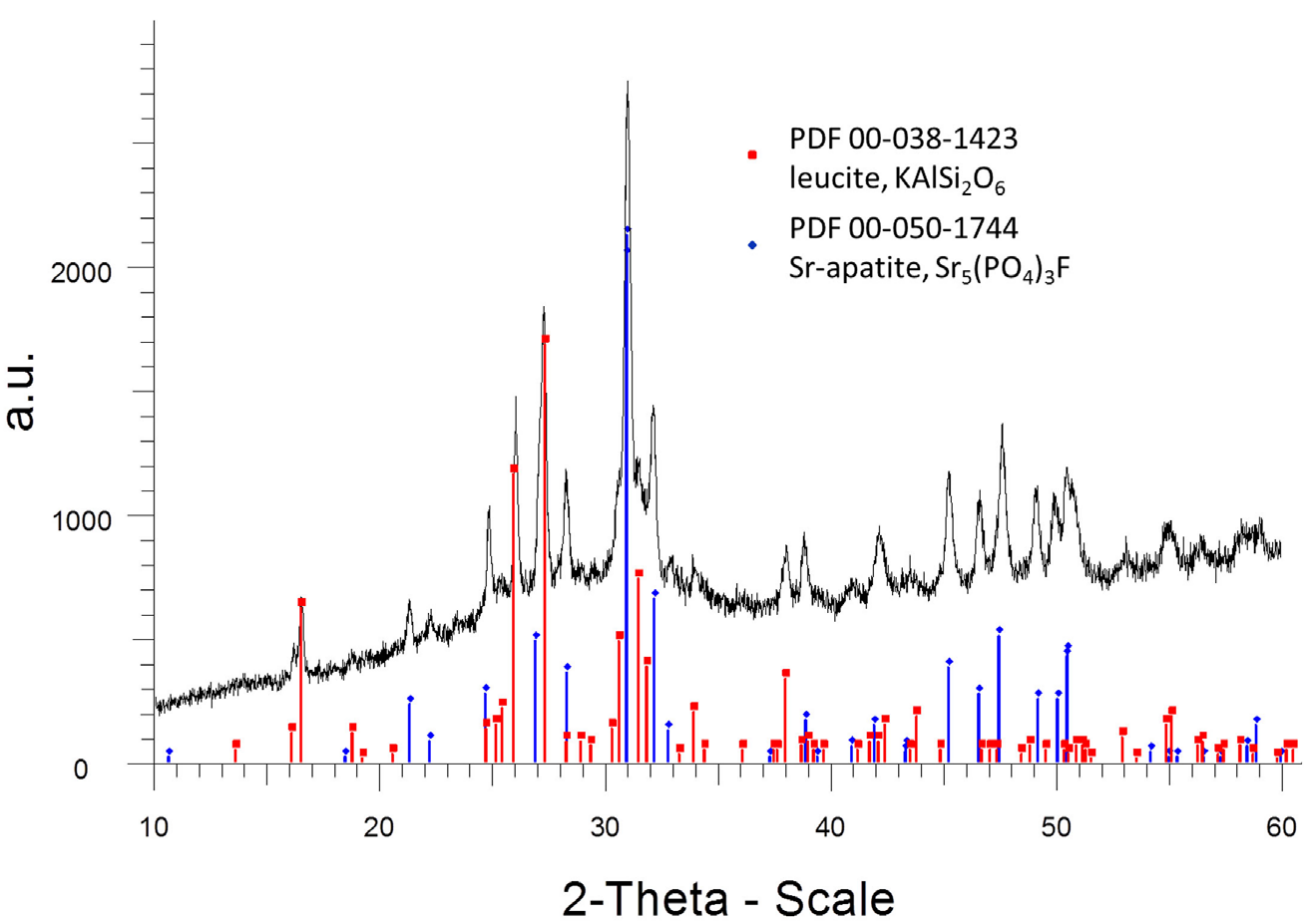
**XRD diagram (room temperature measurement) of glass-ceramic No. 2 after annealing at 1000°C for 1 h**. Two crystal phases, strontium fluoroapatite and leucite were analyzed. The intensities are shown as arbitrary units (a.u.).

The phosphate-rich crystals, however, were not etched by the aqueous HF solution. Instead, the SiO_2_-rich matrix was etched. Therefore, the phosphate crystals ended up protruding from the surface. Based on these SEM and XRD findings, the microstructures of the glass-ceramics No. 1 to 5 show microstructure design according Figures 3–7 with the specific crystal phases demonstrated in Table 2 and Figure 8.

**Table 2:** **Main crystal formation in glass-ceramics at 900 and 1000°C/h**.

Glass-ceramic	Crystal phases after heat treatment of 900°C/h	Crystal phases after heat treatment of 1000°C/h
1	Sr_5_(PO_4_)_3_F, 100 nm in length (Figure 3A)	Sr_5_(PO_4_)_3_F, 800 nm in length and KAlSi_2_O_6_, size 1 μm (holes in Figure 3B)
2	Sr_5_(PO_4_)_3_F, >100 nm in length, minor NaSrPO_4_ (Figure 4A)	Sr(PO_4_)_3_F, KAlSi_2_O_6_ (Figures 4B and 8)
3	Sr_5_(PO_4_)_3_F, CsAlSi_2_O_6_, minor NaSrPO_4_ (Figure 5A)	Sr_5_(PO_4_)_3_F, CsAlSi_2_O_6_, minor NaSrPO_4_ (Figure 5B)
4	Sr_5_(PO_4_)_3_F, RbSi_2_O_6_, minor NaSrPO_4_ (Figure 6A)	Sr_5_(PO_4_)_3_F, RbSi_2_O_6_, minor NaSrPO_4_ (Figure 6B)
5	Ca_5_(PO_4_)_3_F (400–600 nm in length) (Figure 7)	

The crystal phases were determined according to the following XRD reference pattern:

- strontium fluoroapatite (Sr_5_(PO_4_)_3_F): PDF 00-050-1744
- leucite (KAlSi_2_O_6_) phase: PDF 00-038-1423) (see also Figure 8)
- sodium strontium orthophosphate (NaSrPO4): PDF 00-033-1282
- pollucite (CsAlSi_2_O_6_): PDF 00-029-0407
- rubidium leucite (RbSi_2_O_6_): PDF 00-029-1077
- calcium fluoroapatite (Ca_5_(PO_4_)_3_F): PDF 01-074-4390

**Figure 9:**
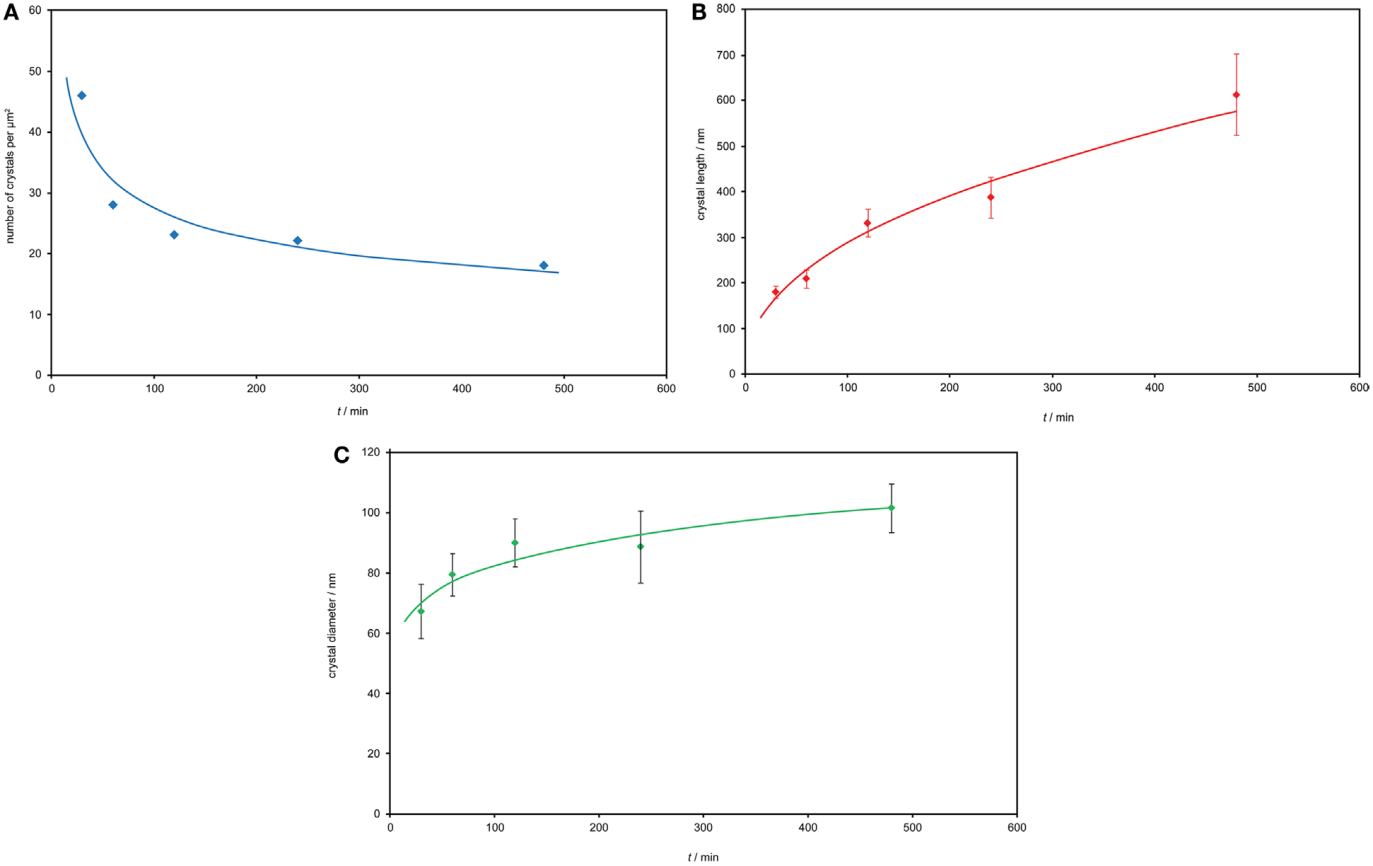
**Crystal growth of needle-like Sr_5_(PO_4_)_3_F at 1000°C in the monolithic glass-ceramic No. 2**. **(A)** Crystal number – time function. **(B)** Crystal length – time relationship. **(C)** Crystal diameter – time function.

**Figure 10:**
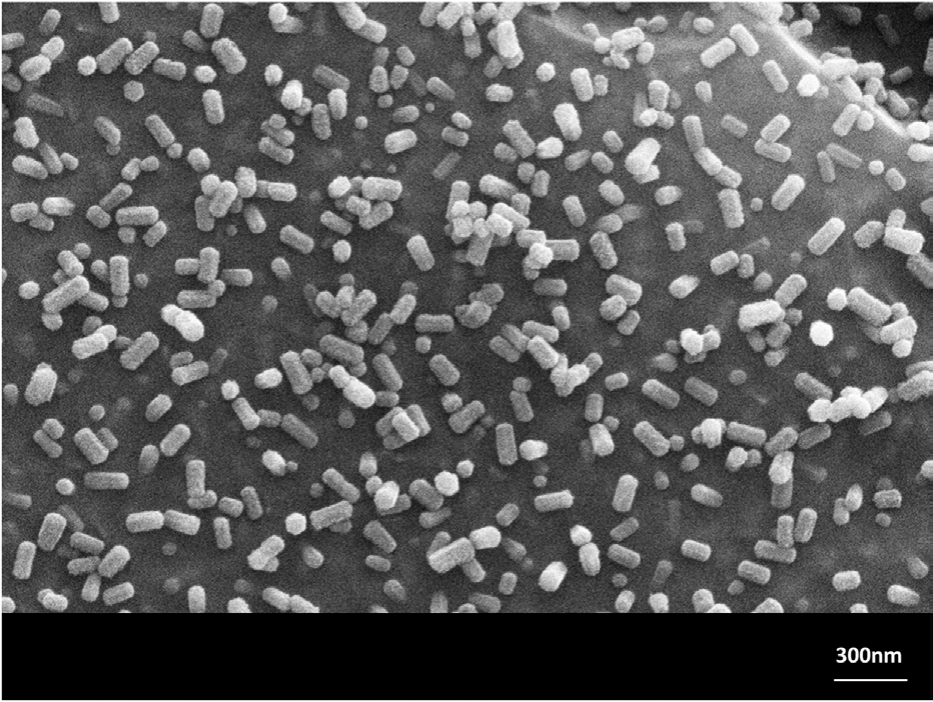
**Microstructure of the monolithic glass-ceramic No. 2 annealed at 1000°C for 30 min**. Needle-like Sr_5_(PO_4_)_3_F crystals were growing. SEM, HF etched surface.

The crystal growth of needle-like Sr_5_(PO_4_)_3_F in the monolithic (no powder compact preparation) glass-ceramic No. 2, annealed at 1000°C, is demonstrated in Figures 9A–C in respect to the number of crystals, the crystal length, and the diameter of the crystals as functions of time (30 min up to 480 min). The Figures 9A–C show characteristics of functional relationships. The first relation involves a decrease of the number of crystals with increasing annealing time (Figure 9A). The second function demonstrates the growth of crystals in length with increasing time (Figure 9B) and the third shows the increase of the crystal diameter with increasing time. The errors are shown as SDs in Figures 9B,C. A typical microstructure of this investigation is shown in Figure 10, demonstrating the microstructure of the needle-like strontium fluoroapatite glass-ceramic after annealing at 1000°C for 30 min.

Processing of the final products of powder compacts proceeded according to the procedure reported in Section “Processing and Chemical Compositions.” According to this procedure, these glass-ceramics prepared from glass frits at 900°C for 1 h, whose microstructures are shown in Figures 3A, 4A, 5A, 6A and 7, were ground and additionally heat treated. The temperatures of the additional heat treatment were also mentioned in Section “Processing and Chemical Compositions.” These temperatures were selected to produce dense powder compacts. Lower temperatures than these did not allow the preparation of dense compacts. During this additional heat treatment, another surface crystallization process was discovered on the surface of the sintered glass grains of glass-ceramics No. 1 and 2: leucite crystals were formed. The proof of such a process could be demonstrated with SEM and XRD studies.

### Properties of the glass-ceramics

#### Thermal Properties

Table 3 shows the CTE values of glass-ceramics No. 1–5. The characteristic values cover a wide range from 10.9 to 19.4 × 10^−6^ K^−1^. The reference sample shows the lowest CTE value of 8.9 × 10^−6^ K^−1^.

**Table 3:** **Main properties of the final glass-ceramics prepared as powder compacts**.

Glass-ceramic	CTE_(100–400°C)_	Optical properties				Radiopacity
	10^−6^⋅K^−1^	L*	a*	b*	CR	% Ratio related to Al
1	13.4	85.2	−1.0	20.10	62.9	350
2	12.6	88.4	−1.3	15.4	59.8	316
3	10.9	94.4	−1.7	6.0	82.5	500–550
4	19.4	94.4	−1.6	5.3	85.1	395
5	8.9	84.2	1.6	11.5	56.9	104

#### Optical Properties

Qualitatively, the glass-ceramics could be characterized as yellowish translucent (glass-ceramic No. 1, 2, and 5) and white with low translucency (glass-ceramic No. 3 and 4). The quantitative values are shown in Table 3. The colors are represented by the L*, a*, b* parameters and the translucency is given with the CR values. The glass-ceramics No. 1 and 2, which were processed from a glass frit at 900°C for 1 h and prepared from this glass-ceramic to form a powder compact at 960°C (glass-ceramic No. 1) and 940°C (glass-ceramic No. 2), were developed as Sr-fluoroapatite – leucite glass-ceramics. These products show the highest translucency in comparison to glass-ceramics No. 3 and 4 (Sr-fluoroapatite – pollucite, and Sr-fluoroapatite – Rb-leucite glass-ceramics).

#### Radiopacity

The radiopacity of the glass-ceramics No. 1 and 2 (Sr-fluoroapatite – leucite glass-ceramics) could be increase by a factor of three in comparison to the reference sample glass-ceramic No. 5 of the Ca-fluoroapatite-type. But a much higher increase of radiopacity could be reached by the development of Sr-fluoroapatite-pollucite type glass-ceramics (glass-ceramic No. 3). The increase of radiopacity in comparison to the reference glass-ceramics represents a factor of five (Table 3).

## Discussion

Vogel (1963, 1985) established the fundamentals of controlled nucleation via glass-in-glass phase separation and used this knowledge to develop glass-ceramics combining a number of different properties. In his studies, he showed the possibilities of glass-in-glass phase separation of different glass-forming systems. His main and unexpected discovery was finding a way to control these processes in glass systems with different glass network formers. His expertise focused on controlling the micro-immiscibility in phosphosilicate glasses. The authors of this paper applied these fundamentals established by Vogel to control the nucleation in a nano-scaled microstructure. That is, the authors of this paper used the nano-glass-in-glass phase separation as a primary stage of the internal nucleation process, which was followed by the internal crystal phase formation and crystal growth of uniform phases in the volume of the glass-ceramics.

In the selected base glasses, the glass-in-glass phase separation proceeded in different ways, according to their chemical nature, which is different to that reported by Hill et al. (2010). In glasses No. 1–4, the process of amorphous glass-in-glass phase separation took place very rapidly during the cooling of the glass melt to room temperature. This led to the precipitation of nano-scaled Sr_5_(PO_4_)_3_F in glasses No. 1 and 2. These nano-sized crystals show a spherical shape, which indicates that crystal formation takes place within an amorphous glass droplet during the cooling of the melt. These crystals grow in needle-like morphology at high temperatures of 1000°C.

The crystal growth process of the needle-like Sr_5_(PO_4_)_3_F follows an Ostwald ripening mechanism as demonstrated in Figures 9A–C for glass-ceramic No. 2 in a monolithic sample. That is, the number of crystals per volume [but determined per area, which can be transformed to volume terms according to DeHoff and Aigeltinger (1970) and Toschev and Gutzow (1967)] decreases as a function of time, while the diameter and length of the crystals grow. Therefore, fundamentals of the theory of Ostwald ripening [reported by Slezov (2009) and Gutzow and Schmelzer (2013)] are fulfilled: The thermodynamically driving force of the process is the reduction of the surface of the crystals by reducing the number of crystals and growing of larger ones. During this process, the total amount of the new phase remains constant but the Gibbs free energy decreases. In more detail, it could be shown that the number of crystals is inversely proportional to the time of annealing (Figure 9A) to some power (−p). In a first approximation, the authors could determine a relationship of the number of crystals per surface area of the sections through the sample, *N*_s_, to the time, *t*, of annealing, according to Eq. 1.

(1) $$N_{\text{s}}\sim t^{-\text{p}}$$

Calculating the curvature of Figure 9A, the best approximation was reached with *p* of −0.306.

But also a calculation of this function with *p* of −3/2 (Eq. 2) showed good results.

(2) $$N\sim t^{-\text{3/2}}$$

Equation 2 is known to describe the number of cluster per volume, *N*, for the coarsening kinetics of ensembles of spherical clusters in the process of kinetic limited growth (Schmelzer, 2015: personal communication), when processes of incorporation at the interface determine the growth rate. This situation might be possible also for the growth of needle-like Sr_5_(PO_4_)_3_F crystals, but a final answer requires more detailed studies, in particular, in performing the determination of the bulk particle number, *N*, from *N*_s_ and appropriately accounting for the shape of the crystallites.

As another point of discussion, a comparison to the findings concerning the growth process of Sr_5_(PO_4_)_3_F crystals should be done with the growth of needle-like Ca_5_(PO_4_)_3_F in a different glass-ceramic system seems as shown by Müller et al. (1999) and Höland et al. (2000). Most importantly, both crystal growth processes appear to follow an Ostwald ripening mechanism. A comparison of the results clearly shows that the number of Sr_5_(PO_4_)_3_F crystals per volume (determined per area) is much higher than that of Ca_5_(PO_4_)_3_F. However, the crystal length of Sr_5_(PO_4_)_3_F with a maximum of approximately 500 nm is much smaller than that of Ca_5_(PO_4_)_3_F, which grew up to 6 μm.

The primary crystal formation in glasses No. 2, 3, and 4 is different to that of glasses No. 1 and 2. The NaSrPO_4_ nano-sized crystals were precipitated during the cooling of the melts of glasses 2, 3, and 4. However, this minor content of NaSrPO_4_ does not influence the formation of Sr_5_(PO_4_)_3_F. The NaSrPO_4_ crystals remain stable in a very low volume fraction, even after annealing at temperatures of up to 1000°C. This phenomenon is similar to that discovered in glass-ceramics precipitation Ca_5_(PO_4_)_3_F and NaCaPO_4_ in a different glass-ceramic system (Höland and Beall, 2012).

The reference glass-ceramic No. 5 contained Ca_5_(PO_4_)_3_F crystals in needle-like morphology. These crystals also grew according to the Ostwald ripening process (Höland and Beall, 2012). However, this glass-ceramic did not contain other crystal phases.

In addition to using the powder compact method for the internal nucleation and crystallization of Sr- or Ca-fluoroapatites, the authors applied this process in order to control surface nucleation and crystallization. This powder compact method involves a glass grain activation process according to the principles of tribochemistry. In this case, the tribochemical reaction was based on the ball milling grinding technology. Activated glass grains initiated the surface nucleation and crystallization of the framework silicates leucite (KAlSi_2_O_6_) or Rb-leucite (RbAlSi_2_O_6_) or pollucite (CsAlSi_2_O_6_). These crystals started to grow on the surface of the glass grains at 1000°C for leucite (glass-ceramic No. 1 and 2) and at 900°C for pollucite (glass-ceramic 3) and Rb-leucite (glass-ceramic No. 4). In a parallel reaction to this twofold nucleation and crystallization of fluoroapatites (via internal mechanisms) and leucite, Rb-leucite, and pollucite crystals (via surface mechanisms), a third reaction was found to take place. This third reaction is the solid-state-liquid-state sintering of the crystallized glass grains to form a glass-ceramic powder compact. This sintering involves a diffusion process in which the glass matrix is the main component. To produce a densely sintered glass-ceramic compact, these processes of powder compact formation took place at temperatures that were higher than those used for the formation of the glass-ceramic, that is, above 900°C. The reaction time was very short but led to the additional formation of leucite crystals. Therefore, because of their crystal phases, the four glass-ceramics have to be characterized as follows:

*Glass-ceramic No. 1*: Sr_5_(PO_4_)_3_F–leucite, KAlSi_2_O_6_,
*Glass-ceramic No. 2*: Sr_5_(PO_4_)_3_F–leucite, KAlSi_2_O_6_–NaSrPO_4_,
*Glass-ceramic No. 3*: Sr_5_(PO_4_)_3_F–pollucite, CsAlSi_2_O_6_–NaSrPO_4_,
*Glass-ceramic No. 4*: Sr_5_(PO_4_)_3_F–Rb-leucite, RbAlSi_2_O_6_–NaSrPO_4_.

The final powder compact glass-ceramics show different properties according to their chemical nature, crystal phase formation, and the very special design of their microstructure.

The main aim of this study was the development of glass-ceramics with a high level of radiopacity. The radiopacity was controlled by precipitating Sr-fluoroapatite crystals instead of Ca-fluoroapatites, and by incorporating Y^3+^ ions into the glass structure. Therefore, glass-ceramics No. 1 and 2 show much higher radiopacities than the reference glass-ceramic No. 5. An additional effect of increasing the radiopacity could be realized by adding Rb^+^ and Cs^+^ ions to the glass-ceramic. This incorporation of Rb^+^ and Cs^+^ ions in the glass-ceramics led to the crystallization of Rb-leucite and pollucite, respectively. Based on these findings, the pollucite-type glass-ceramic No. 3 containing Sr-fluoroapatite crystals showed the highest radiopacity.

In addition, it was established that the CTE values could be controlled. Glass-ceramic No. 4, characterized as Sr-fluoroapatite–Rb-leucite glass-ceramic, with nano-sized phases of NaSrPO_4_, showed the highest CTE of all the studied glass-ceramics. The optical properties (translucency and color) of all the glass-ceramics discussed provide a basis on which further biomaterials for dental applications can be developed.

## Conclusion

The authors conclude that glass-ceramics derived from the chemical SiO_2_–Al_2_O_3_–Y_2_O_3_–SrO–Na_2_O–K_2_O/Rb_2_O/Cs_2_O–P_2_O_5_–F system can be developed as powder compact products. The ­processes of nucleation and crystallization were utilized by applying twofold mechanisms of internal and surface reactions. Based on these fundamentals, leucite-type, Rb-leucite-type, and pollucite-type glass-ceramics containing needle-like Sr_5_(PO_4_)_3_F crystals were developed.

In respect to the primary nano-sized crystal formation, the findings allow the conclusion that the formation of these spherical crystals of disordered Sr_5_(PO_4_)_3_F or NaSrPO_4_ is based on glass-in-glass phase separation. Nano-sized Sr_5_(PO_4_)_3_F turns into needle-like Sr_5_(PO_4_)_3_F. The growth of crystals follows an Ostwald ripening mechanism. The authors conclude that this process give a basis of tailor-made processing of glass-ceramics exhibiting controlled optical properties, especially controlled translucency and brightness.

Nevertheless, the NaSrPO_4_ nano-phase was shown to have no epitaxial effect on Sr_5_(PO_4_)_3_F formation. This is similar to the phenomenon discovered in KAlSi_2_O_6_–Ca_5_(PO_4_)_3_F–NaCaPO_4_ glass-ceramics.

In summary, the authors conclude that twofold crystallization processes can be successfully applied to develop glass-ceramic biomaterials which combine different properties, such as specific radiopacity values, CTEs, and other optical characteristics. The main conclusion in respect of developing glass-ceramics showing high radiopacity is the influence of the large atoms/ions and their atomic/ionic mass of Cs^+^ and Rb^+^ over other alkali ions. Also, the increase of radiopacity of all the Sr-apatite glass-ceramics in comparison to Ca-fluoroapatite galss-ceramics should be based on the larger ion and greater atomic/ionic mass of Sr^2+^ over Ca^2+^ within the apatite crystal structure.

## Conflict of Interest Statement

The research was done for a company: Ivoclar Vivadent AG.
